# G9A promotes gastric cancer metastasis by upregulating ITGB3 in a SET domain-independent manner

**DOI:** 10.1038/s41419-018-0322-6

**Published:** 2018-02-15

**Authors:** Lei Hu, Ming-de Zang, He-xiao Wang, Bao-gui Zhang, Zhen-qiang Wang, Zhi-yuan Fan, Huo Wu, Jian-fang Li, Li-ping Su, Min Yan, Zhi-qiang Zhu, Qiu-meng Yang, Qiang Huang, Bing-ya Liu, Zheng-gang Zhu

**Affiliations:** 1 Department of Surgery, Shanghai Key Laboratory of Gastric Neoplasms, Shanghai Institute of Digestive Surgery, Ruijin Hospital, Shanghai Jiao Tong University School of Medicine, 200025 Shanghai, People’s Republic of China; 2 Department of General Surgery, Affiliated Provincial Hospital of Anhui Medical University, 230001 Hefei, People’s Republic of China; 3 Affiliated Hospital of Jining Medical University, 272000 Jining, People’s Republic of China

## Abstract

Tumor metastasis is the leading cause of death in patients with advanced gastric cancer (GC). Limited therapeutic regimens are available for this condition, which is associated with a poor prognosis, and the mechanisms underlying tumor metastasis remain unclear. In the present study, increased histone methyltransferase G9A expression in GC tissues correlated with advanced stage and shorter overall survival, and in vitro and in vivo experiments revealed that G9A promoted tumor invasion and metastasis. Moreover, we observed that Reg IV induced G9A via the p-ERK/p-SP1 pathway. SP1 directly binds the G9A promoter and enhances G9A expression, and upregulated G9A then forms a transcriptional activator complex with P300 and GR, thereby promoting ITGB3 expression induced by dexamethasone (DEX) and contributing to GC metastasis. However, the G9A-mediated increase in ITGB3 expression was not dependent on the SET domain and methyltransferase activity of G9A. This study demonstrates that G9A is an independent prognostic marker and promotes metastasis in GC, thus suggesting that it may be a tumor biomarker and potential therapeutic target in GC.

## Introduction

Gastric cancer (GC) is a malignant tumor derived from gastric epithelial cells and is associated with high morbidity and mortality^1^. Metastasis and invasion are the most important features of GC, and approximately 50% of metastases are peritoneal metastases, which are associated with a poor prognosis^2^. Because metastatic cells can affect multiple vital organs within the abdomen, the average survival time of patients with peritoneal metastases is <6 months^3^. Unfortunately, the molecular mechanisms by which advanced GC progress to metastasis remain poorly understood. In view of the detrimental influence of advanced metastasis on survival, exploring the molecular mechanisms of metastasis and developing treatment or prevention strategies is of great significance.

G9A, also known as EHMT2, is a nuclear histone lysine methyl-transferase belonging to the Su(var)3-9 family, which plays a primary role in catalyzing the monomethylation and dimethylation of H3K9 (H3K9me1 and H3K9me2) in euchromatin^4^. G9A is involved in autophagy, cell differentiation, embryonic development, cognitive and adaptive behavior, adipogenesis, and other biological processes^5–9^. Moreover, recent studies have indicated that G9A expression is elevated in many types of human cancers, including breast cancer^10^, ovarian cancer^11^, head and neck squamous cell carcinoma^12^, lung cancer^13^ and endometrial cancer^14^, and G9A has been reported to promote both cell proliferation and metastasis in cancers. For example, G9A-specific small interfering RNAs (siRNAs) result in a DNA damage response and inhibit the proliferation of colorectal cancer cells^15^. Furthermore, BIX-01294, an inhibitor of G9A methyltransferase activity, induces autophagy-dependent cell death via G9A dysfunction and intracellular reactive oxygen species accumulation in breast and colon cancer cells^16^. G9A also exhibits methyl-transferase activity and concomitantly represses the downstream effector Ep-CAM, thereby promoting the invasion step of the invasion–metastasis cascade in lung cancer^13^. Accordingly, the knockdown of G9A expression suppresses the peritoneal metastasis of ovarian cancer^11^, thus prompting us to study the role of G9A in GC peritoneal metastasis. G9A promotes the methylation of gene promoter region histones or DNA via two different transcription-inhibitory mechanisms^17,18^. Alternatively, G9A can act as a scaffold protein promoting the expression of transcription activators, thereby activating gene transcription^19,20^.

Accumulating evidence indicates that G9A is associated with tumor development, but the function of G9A in GC remains poorly understood. In the present study, we characterized the expression of G9A and evaluated the correlation between G9A expression and the clinicopathological features of GC. Moreover, the biological functions of G9A were investigated by silencing or restoring G9A expression in GC cells and animal models. Our results indicated that G9A expression strongly correlated with the metastatic properties of GC and may serve as a potential therapeutic target.

## Results

### Expression of G9A and its clinicopathologic significance in patients with GC

**Fig. 1:**
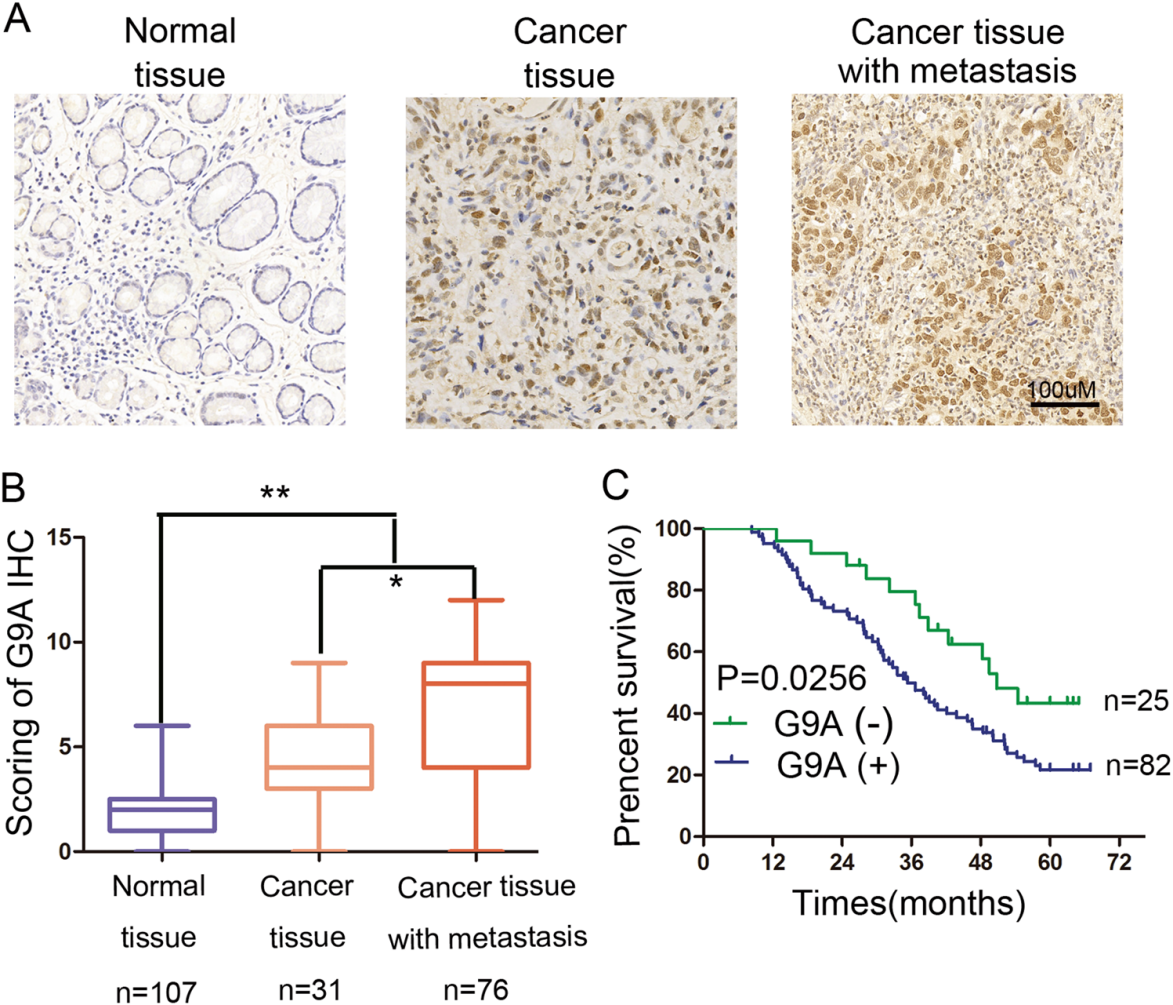
Expression of G9A and its clinical significance in patients with GC. **a** Immunohistochemical analysis of G9A in normal gastric mucosa, GC tissues, and GC tissues from patients with metastasis. **b** Plots depicting scores according to the immunohistochemical expression of G9A in normal gastric mucosa, GC tissues, and GC tissues from patients with metastasis. **c** Kaplan–Meier survival curves for patients with GC according to G9A staining. Patients with positive G9A staining had a significantly poorer prognosis than those with negative staining. Each value is the mean ± SD of three experiments. *P < 0.05, **P < 0.01

To explore the potential roles of G9A in GC development, we investigated G9A mRNA expression in the Oncomine database (www.oncomine.org/) and TCGA database (https://cancergenome.nih.gov/). We found that the G9A transcript level was significantly increased in GC tissues compared with matched non-tumor tissues, and the expression of G9A did not differ in LAUREN subtypes of GC (Figure S1A, B). We also analyzed the relationship between G9A expression and molecular subtypes in TCGA datasets and found that G9A was highly expressed in subtypes EBV and CIN but lower in subtypes MSI and GS (Figure S1C). We then measured G9A protein expression in 107 pairs of GC and normal tissues by immunohistochemistry (IHC). As shown in Fig. 1a, b, G9A staining was mainly detected in the nuclei of cells in GC tissues, and almost no G9A staining was observed in matched non-tumor tissues. Surprisingly, we found that the expression of G9A was much higher in primary cancer tissues from patients with metastatic GC than in those from patients without metastases. We next investigated the relationship between G9A expression and the clinicopathologic features of GC and found that G9A overexpression was associated with lymph node metastasis (*P* = 0.034) and Tumor, Node, Metastasis (TNM) stage (*P* = 0.003) but not with other clinicopathologic factors, including tumor differentiation, the extent of invasion, and tumor location (Table 1). Kaplan–Meier survival analysis indicated that the survival rate (from the time of surgery) of patients with positive staining was significantly lower than that of patients with negative staining (*P* = 0.0256, Fig. 1c). Moreover, multivariate analyses indicated that G9A expression was an independent prognostic factor of 5-year overall survival in patients with GC (*P* = 0.033, Table 2). These results suggested that upregulated G9A expression is associated with GC development.

**Table 1:** Association between G9A expression and clinicopathological factors of gastric cancer patients

Variables	Number (*n* = 107)	G9A immunostaining		*P*
		Positive (*n* = 82)	Negative (*n* = 25)	
Gender				0.954
Male	69	53	16	
Female	38	29	9	
Age(years)				0.169
≥60	72	58	14	
<60	35	24	11	
Tumor size				0.931
≤5cm	35	27	8	
>5cm	72	55	17	
Differentiation				0.140
Well to moderate	39	33	6	
Poor	68	49	19	
Lauren subtype				
Intestinal type	58	45	13	0.941
Diffuse type	34	26	8	
Mixed type	15	11	4	
Extent of invasion				0.579
T1+T2	42	31	11	
T3+T4	65	51	14	
Lymph node involvement				0.034
Absence	31	21	10	
Presence	76	61	15	
Distant metastasis				0.922
Absence	91	78	23	
Presence	6	4	2	
TNM stage				0.003
I+II	38	23	15	
III+IV	69	59	10	

Note: Positive G9A expression included all positive cases, such as weak and strong

**Table2:** Multivariate Cox regression analysis of the overall survival of 107 GC patients

Variables	Overall survival	
	*P*	HR (95% CI)
Gender	0.269	0.764 (0.475–1.230)
Age	0.917	1.026 (0.631–1.668)
Tumor size	0.459	0.836 (0.520–1.344)
TNM stage	0.972	0.991 (0.603–1.630)
G9A expression	0.033	1.950 (1.057–3.600)

### G9A promotes GC cell migration and invasion

**Fig. 2:**
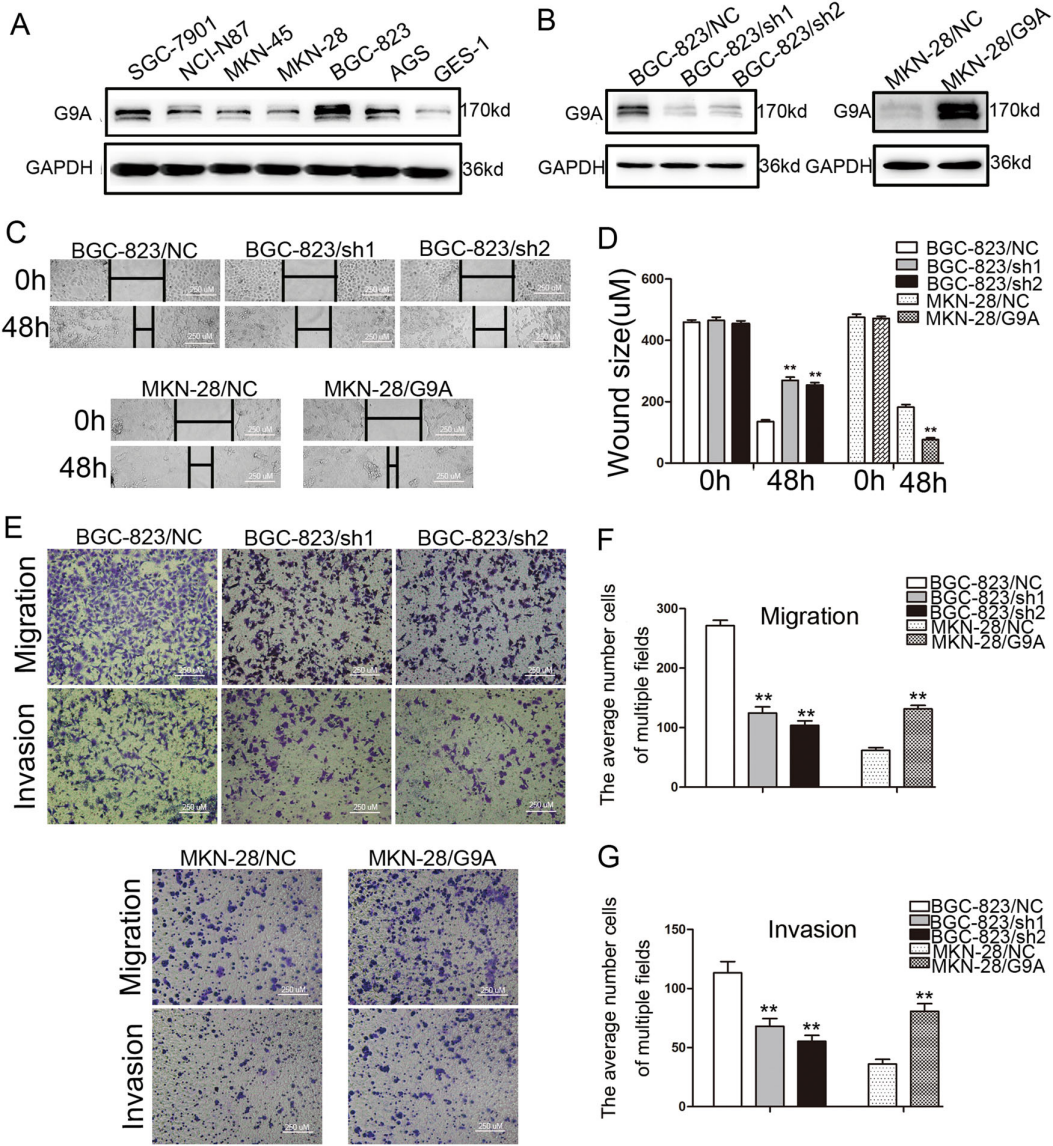
Effects of G9A on wound healing, migration, and invasion ability in GC cells (200×). **a** G9A expression in six GC cell lines and one immortalized gastric epithelial cell line, as detected by western blotting. **b** BGC-823 and MKN-28 cells transfected with sh-G9A lentivirus and G9A overexpression lentivirus, respectively, were subjected to western blotting. **c** Representative images from BGC-823/sh-G9A, MKN-28/G9A cells, and their control groups were recorded 0 and 48 h after scratching of the cell surface. **d** The relative distances between the wound edges of GC cells at 0 and 48 h. **e** The migration and invasive behavior were evaluated by using a Transwell chamber with or without Matrigel after knockdown or overexpression of G9A in BGC-823 or MKN-28 cells. **f** Histograms showed the average number of migrating cells in multiple fields. **g** Histograms showed the average number of invading cells in multiple fields. Each value is the mean ± SD of three experiments. **P < 0.01

G9A expression was measured in GC cells and the immortalized gastric epithelial cell line GES-1 by using quantitative real-time polymerase chain reaction (qRT-PCR) and western blot assays. As shown in Fig. 2a and Figure S1C, G9a was highly expressed in GC cell lines compared with GES-1 cells. Among the six GC cell lines, the expression of G9A was higher in BGC-823, SGC-7901, and AGS cells but lower in MKN-28, MKN-45, and NCI-N87 cells. Next, G9A was knocked down in BGC-823 and SGC-7901 cells (BGC-823/shG9A, SGC-7901/shG9A) and upregulated in MKN-28 and MKN-45 cells (MKN-28/G9A, MKN-45/G9A) by lentiviral transfection (Fig. 2b and Figure S1D-G).

To investigate the effect of G9A on GC cell motility, a wound-healing assay was performed. We observed that G9A knockdown significantly inhibited the wound-healing ability of BGC-823 cells after 48 h (*P* < 0.01, Fig. 2c, d). In contrast, the distance between the wound edges of MKN-28 cells was markedly shorter when exogenous G9A was upregulated by lentiviral transfection (*P* < 0.01, Fig. 2c, d). We then performed Transwell assays to investigate the influence of G9A on GC cell migration and invasiveness. Similarly, we found that the downregulation of G9A significantly decreased the migration and invasion of BGC-823 cells, whereas the overexpression of G9A increased MKN-28 cell migration and invasion (*P* < 0.01, Fig. 2e–g). Moreover, these findings were further confirmed in SGC-7901 and MKN-45 cells (Figure S2).

### G9A promotes the peritoneal metastasis-related traits of GC in vitro

**Fig. 3:**
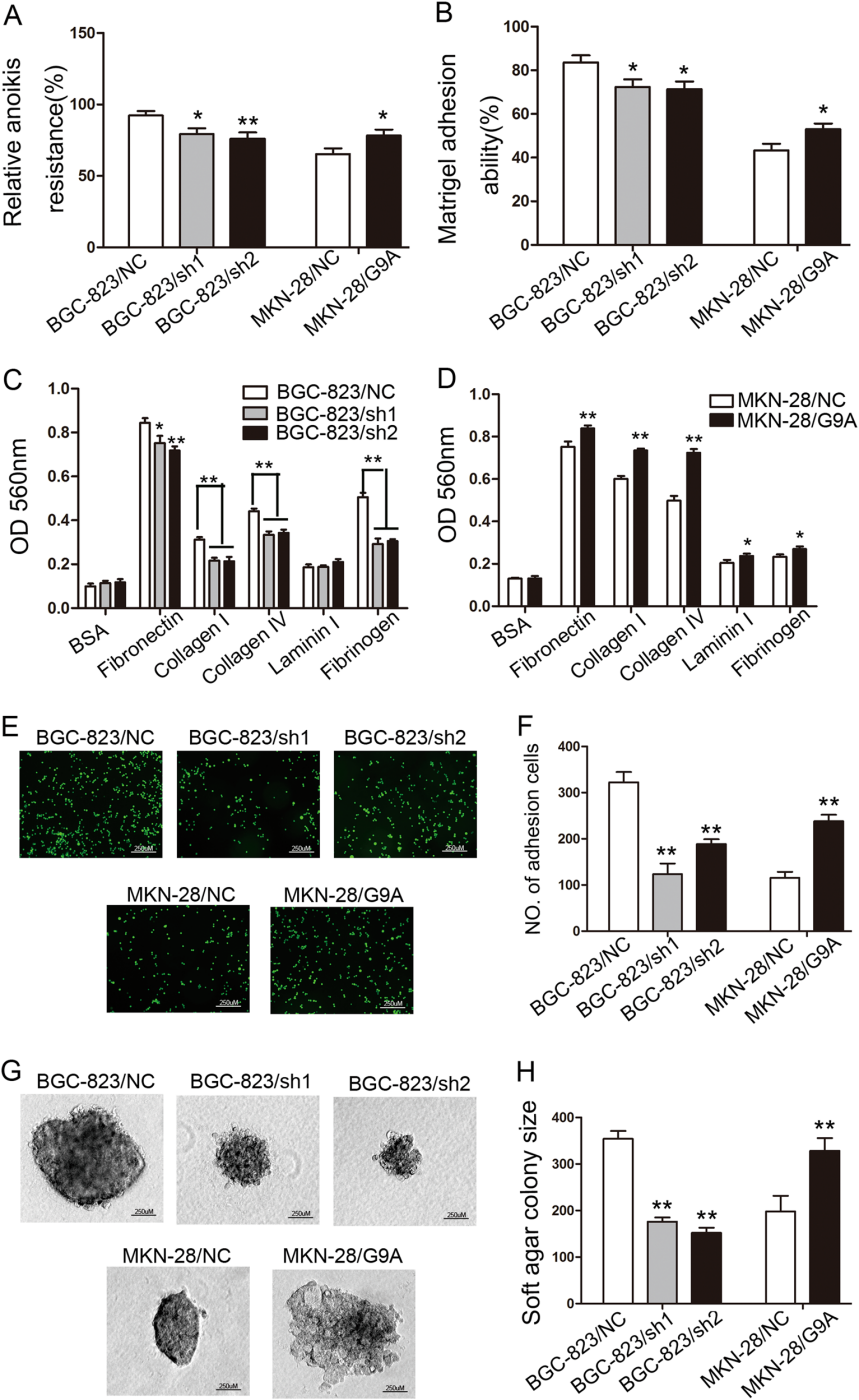
G9A promotes the peritoneal metastasis of GC cells. **a** Anoikis assays of BGC-823 and MKN-28 cells infected with a specific sh-G9A lentivirus and G9A overexpression lentivirus, as indicated. **b** Adhesion of GC cells in which G9A was downregulated or overexpressed to Matrigel-coated surfaces. **c**, **d** The percentage of GC cells in which G9A was knocked down (**c**) and overexpressed (**d**) that adhered to plates coated with different ECM components after 30 min of incubation were quantified on the basis of the OD at 560 nm. **e**, **f** Representative images (**e**) and the number of cells (**f**) that adhered to murine peritoneum after 60 min of incubation. **g**, **h** Representative images (**g**) and colony size of cells (**h**) in soft agar assay after 14 days of incubation. Each value is the mean ± SD of three experiments. *P < 0.05, **P < 0.01

When GC cells pass through the serous layer into the abdominal cavity, they enter a detached environment. Cell anoikis was increased in G9A knockdown cells, as compared with control cells, whereas G9A upregulation significantly promoted cell survival, thus suggesting that the presence of G9A counteracts anoikis (*P* < 0.05, Fig. 3a). Because adhesion to the peritoneum is the prerequisite for peritoneal metastasis, the effect of G9A on the adhesive behavior of GC cells was evaluated by using plates coated with Matrigel. When G9A expression was suppressed in BGC-823 cells, adhesion to Matrigel meaningful decreased (*P* < 0.05, Fig. 3b). To further corroborate the effect of G9A on cell adhesion, adhesion assays using plates coated with different extracellular matrix (ECM) components were performed. As expected, the cells exhibited lower adhesion rates to fibronectin, collagen I, collagen IV, and fibrinogen when G9A was downregulated (*P* < 0.05, Fig. 3c). To mimic the in vivo environment, we also used murine peritoneum as a substrate in the cell adhesion assay, and the results further confirmed that the downregulation of G9A decreased the adhesiveness of GC cells (*P* < 0.01, Fig. 3e, f). In contrast, MKN-28/G9A cells, in which G9A was upregulated, adhered better to Matrigel, ECM components, and murine peritoneum than control cells (*P* < 0.05, Fig. 3b, d–f). GC cells undergo anchorage-independent growth in the abdominal cavity, and we simulated this growth environment with a soft agar colony-formation assay. We found that G9A-depleted GC cells grew smaller colonies on soft agar than did control cells, whereas G9A-overexpressing GC cells grew larger colonies on soft agar than did control cells (*P* < 0.01, Fig. 3g, h and Figure S3). In addition to BGC-823 and MKN-28 cells, these experiments produced similar results in SGC-7901 and MKN-45 cells (Figure S4).

### G9A promotes the peritoneal metastasis of GC in vivo

**Fig. 4:**
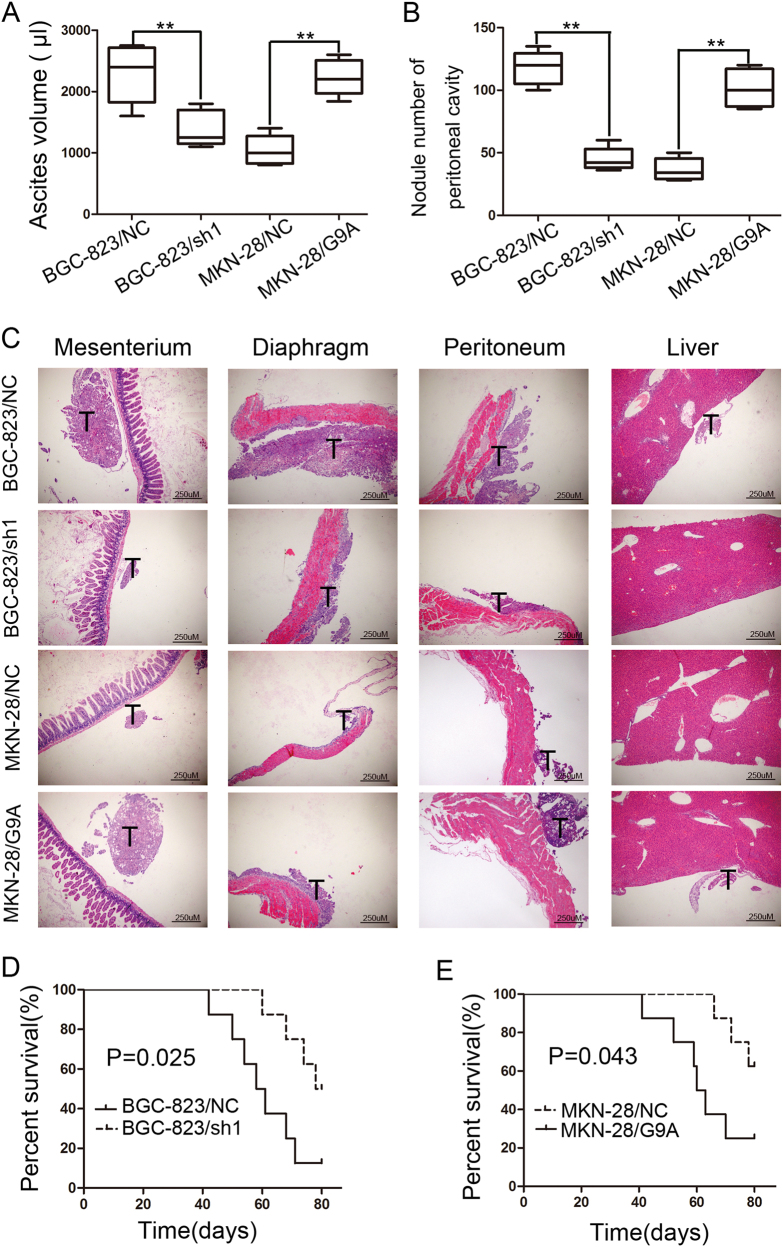
Functional effects of G9A overexpression/knockdown on the peritoneal dissemination potential of GC cells in vivo. **a** Box and whisker plot of the ascite volumes collected from the control groups and G9A knockdown/overexpression groups (each group *n* = 8). **b** Box and whisker plot of the disseminated tumor number collected from the controls and G9A knockdown/overexpression groups (each group *n* = 8). **c** The histological appearance of disseminated tumors in the abdominal cavities was assayed via H&E staining from mice in the control groups and G9A knockdown/overexpression groups (each group *n* = 8). **d**, **e** Survival curves for mice. BGC-823/sh1 (**d**) and MKN-28/G9A (**e**) cells compared with their control groups (each group *n* = 8). *n* number; *T* tumor. Each value is the mean ± SD of three experiments. **P < 0.01

The effect of G9A on peritoneal metastasis was evaluated in vivo. Mice injected with BGC-823/sh1 cells exhibited less ascite formation 80 days after inoculation compared with mice injected with BGC-823/NC cells (*P* < 0.01, Fig. 4a). The opposite results were observed between MKN-28/G9A and MKN-28/NC cells (*P* < 0.01, Fig. 4a). As expected, animal experiments showed that silencing G9A led to significantly fewer visible peritoneal nodules in BGC-823 cells, whereas upregulation of G9A markedly increased visible peritoneal nodules in MKN-28 cells (*P* < 0.01, Fig. 4b). A histological analysis of the xenografts confirmed that BGC-823/NC and MKN/G9A cells developed larger tumors with more organ involvement in the abdominal cavity (peritoneum, mesentery, diaphragm, and liver) (Fig. 4c and Figure S5). Moreover, mice inoculated with BGC-823/sh1 cells exhibited longer overall survival than mice injected with BGC-823/NC cells (*P* = 0.025, Fig. 4d). In contrast, upregulation of G9A markedly decreased the overall survival time of mice in vivo (*P* = 0.043, Fig. 4e). In conclusion, these in vivo experiments suggested that G9A is essential for promoting the peritoneal metastasis of GC cells.

### Reg IV promotes G9A expression via phospho-extracellular signal–regulated kinase (p-ERK)/p-SP1 signaling

**Fig. 5:**
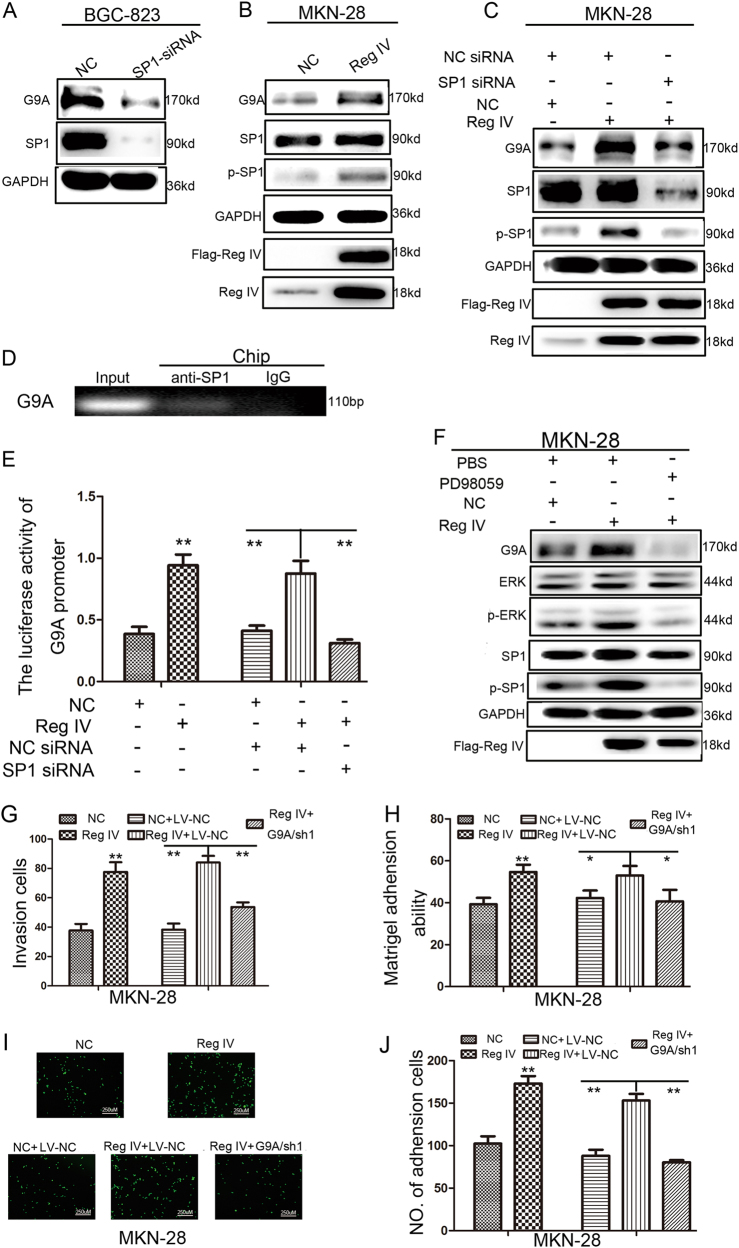
Reg IV promotes G9A expression via p-ERK/p-SP1 signaling. **a** The G9A and SP1 protein levels in BGC-823 cells treated with SP1 siRNA were measured by western blotting. **b** G9A and p-SP1 levels in MKN-28 cells transfected with Reg IV. **c** G9A and p-SP1 changes at the protein level in MKN-28 cells transfected with SP1 siRNA, Reg IV siRNA, or both SP1 and Reg IV siRNA. **d** ChIP assay for the binding of SP1 to the G9A promoter in BGC-823 cells. **e** G9A promoter activity changes in MKN-28 cells transfected with SP1 siRNA, Reg IV siRNA, or both SP1 and Reg IV siRNA. **f** G9A and p-SP1 changes at the protein level in MKN-28 cells treated with PD98059, Reg IV, or both PD98059 and Reg IV. **g** Histograms showing the numbers of invading GC cells transfected with Reg IV overexpression lentivirus, G9A interference lentivirus, or both Reg IV overexpression lentivirus and G9A interference lentivirus. **h** Adhesion of GC cells transfected with Reg IV overexpression lentivirus, G9A interference lentivirus, or both Reg IV overexpression lentivirus and G9A interference lentivirus to Matrigel-coated surfaces. **i**, **j** Representative images (**i**) and the number of GC cells (**j**) adherent to murine peritoneum after cells were transfected with Reg IV overexpression lentivirus, G9A interference lentivirus, or both Reg IV overexpression lentivirus and G9A interference lentivirus. Each value is the mean ± SD of three experiments. *P < 0.05, **P < 0.01

Several SP1-binding sites were identified on the promoter of G9A, and the knockdown of SP1 in BGC-823 cells markedly decreased G9A expression (Fig. 5a). On the basis of these findings and those of our previous study showing that Reg IV activates SP1^21^, we transfected Reg IV into MKN-28 cells. These cells exhibited significantly increased G9A and p-SP1 protein levels (Fig. 5b). Furthermore, the depletion of SP1 expression abrogated the induction of G9A by Reg IV (Fig. 5c), and G9A suppression also attenuated the effects of Reg IV on adhesion and invasion (*P* < 0.05, Fig. 5g–j). Moreover, a chromatin immunoprecipitation (ChIP) assay in BGC-823 cells demonstrated that SP1 directly bound to the G9A promoter and promoted G9A transcription (Fig. 5d). This finding was confirmed by the co-transfection of Reg IV and the G9A promoter, which increased luciferase activity in GC cells, whereas the depletion of SP1 expression attenuated the increase in luciferase activity in MKN-28 cells (*P* < 0.01, Fig. 5e). ERK, an upstream molecular of SP1, is also activated by Reg IV. Therefore, we investigated its role in G9A induction by examining p-SP1. As shown in Fig. 5f, pretreatment with the ERK inhibitor PD98059 attenuated increases in the G9A and p-SP1 protein levels. These results demonstrated that the p-ERK/p-SP1 pathway is critical for the link between Reg IV and G9A.

### G9A non-SET domain recruitment is sufficient for GR-induced ITGB3 expression

**Fig. 6:**
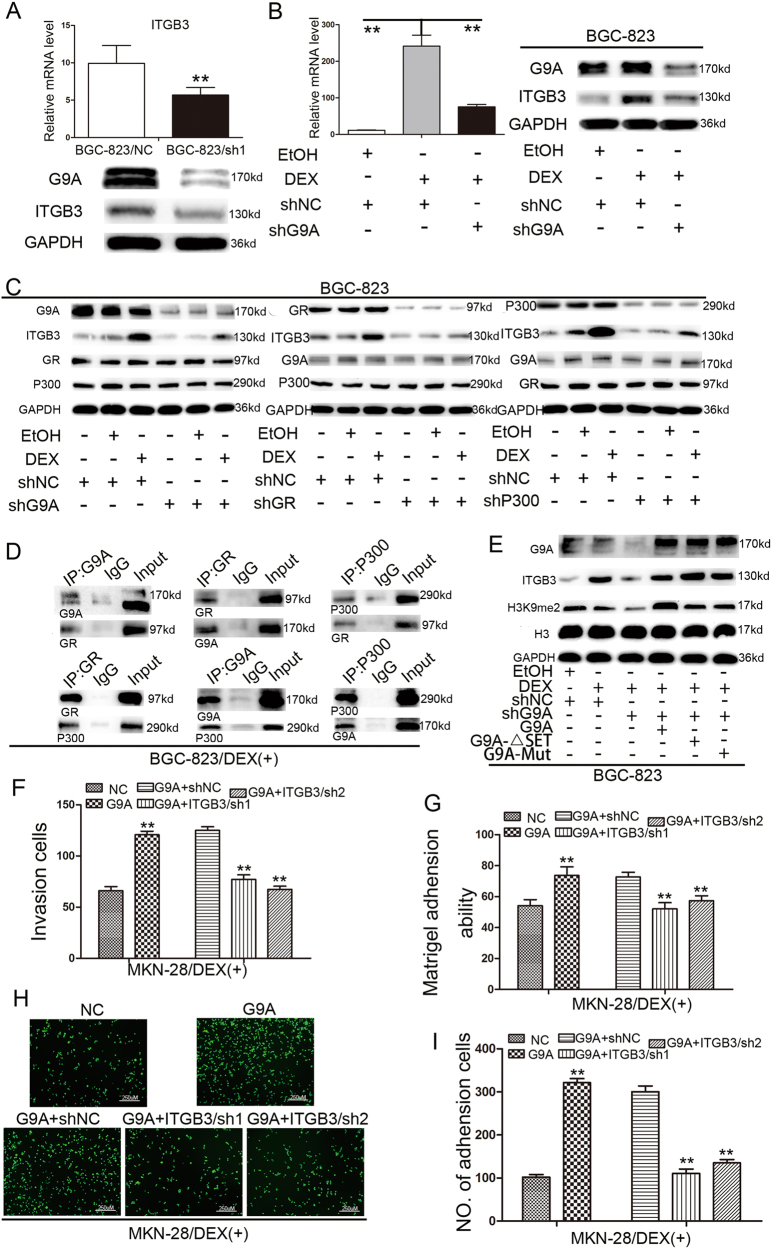
G9A participates in GR hormone-mediated ITGB3 induction via the GR/P300/G9A complex. **a** ITGB3 changes at the RNA and protein levels in BGC-823 cells treated with shG9A lentivirus. **b** ITGB3 changes at the RNA and protein levels in GC cells treated with shG9A lentivirus, DEX, or shG9A lentivirus and DEX. **c** ITGB3 changes at the protein level in GC cells treated with shG9A/shNC lentivirus, shGR/shNC vector, or shP300/shNC vector in the presence or absence of DEX. EtOH treatment was used as a control for DEX. **d** Endogenous G9A, GR, and P300 were immunoprecipitated from BGC-823 cells stimulated with DEX and examined by western blotting. **e** ITGB3 and H3K9me2 changes at the protein level in GC cells transfected with shG9A/shNC lentivirus, G9A/G9A-ΔSET/G9A-mut vector, and DEX/EtOH treatment alone or in combination. **f** Histograms showing the number of invading DEX-stimulated GC cells transfected with shITGB3 or shNC vector. **g** Adhesion of ITGB3 knockdown GC cells stimulated with DEX to Matrigel-coated surfaces. **h**, **i** Representative images (**i**) and the number of GC cells (**k**) adherent to murine peritoneum after treatment with shITGB3 or shNC vector and DEX stimulation. Each value is the mean ± SD of three experiments. **P < 0.01

**Fig. 7:**
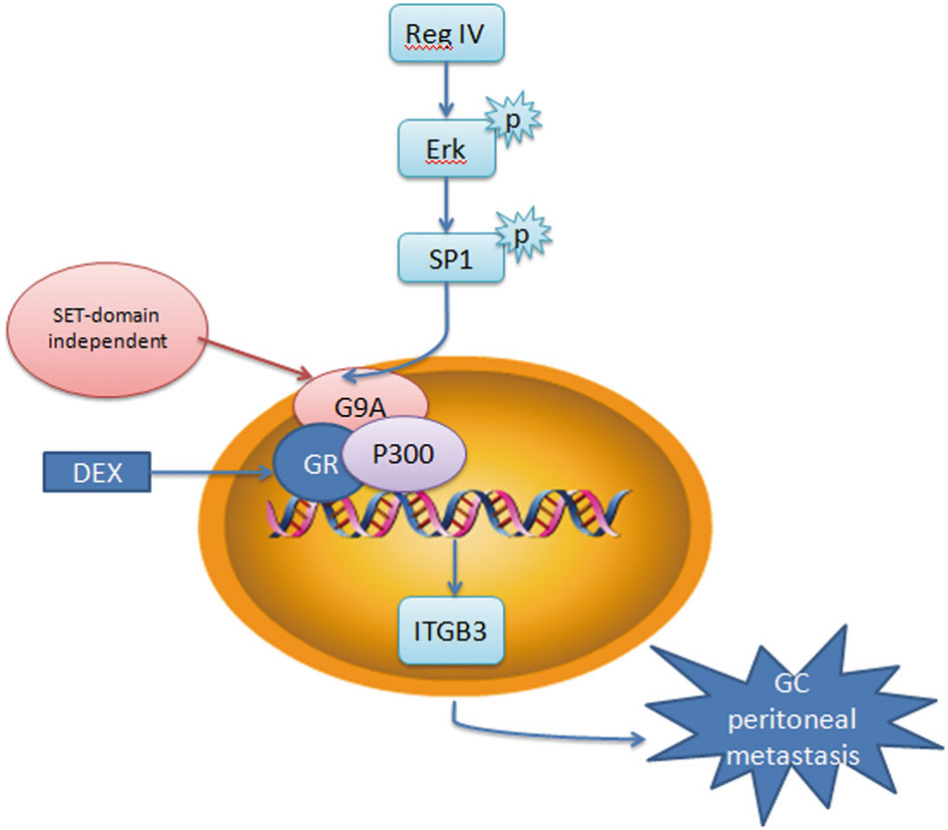
Model of the possible mechanism underlying G9A-induced metastasis in GC and the regulatory mechanism of G9A. Activation of p-ERK/p-SP1 by Reg IV in GC cells leads to the overexpression of G9A and the formation of a complex with P300 and GR in response to glucocorticoid stimulation, which results in increased ITGB3 expression and promotes the peritoneal metastasis of GC cells

ITGB3, a member of the integrin family, plays an important role in the peritoneal metastasis of GC^22^. Therefore, we examined the ability of G9A to regulate ITGB3 expression in GC cells. Specifically, silencing G9A downregulated ITGB3 expression (Fig. 6a). ITGB3 is also a GR target gene in the presence of dexamethasone (DEX) stimulation, and G9A can bind to GR to transcriptionally activate genes^19^. Thus we investigated the effect of G9A on ITGB3 expression. The knockdown of G9A attenuated DEX-induced ITGB3 expression (Fig. 6b). G9A usually forms a coactivator complex with nuclear receptors, thereby regulating gene expression. Accordingly, the effect of knocking down G9A, GR, or P300 on the expression of ITGB3 was examined in the presence or absence of DEX induction (Figure S6), and the results indicated that G9A, GR, and P300 are all involved in the DEX-induced expression of ITGB3 (Fig. 6c). To further illustrate the interaction of G9A, GR, and P300, we used a co-immunoprecipitation (Co-IP) assay, which confirmed that G9A, GR, and P300 interact with one another, thus forming a coactivator complex after DEX stimulation (Fig. 6d), whereas the Co-IP signal was negative or weak in the absence of DEX (Figure S7). Importantly, to examine the need for the G9A SET structure domain in DEX-induced ITGB3 expression, we transfected G9A knockdown cells with a G9A vector with methyltransferase activity and a G9A-△SET/G9A-mut vector without methyl-transferase activity. Both constructs rescued ITGB3 expression after DEX stimulation, but only the G9A vector rescued H3K9me2 expression, thus indicating that the participation of G9A in DEX-induced ITGB3 expression does not depend on the SET domain (Fig. 6e). Moreover, the invasiveness and adhesion of MKN-28/G9A cells were both attenuated after transfection with ITGB3 short hairpin RNA (shRNA) (*P* < 0.01, Fig. 6e). Thus the non-SET region of G9A was both sufficient and necessary for the G9A coactivator function in DEX-induced expression of ITGB3. On the basis of these data, we propose that G9A plays an important role in the process of peritoneal metastasis via the Reg IV/p-ERK/p-SP1/G9A complex/ITGB3 pathway (Fig. 7).

## Discussion

Considerable efforts have been made to explore the function of G9A in a variety of cancer tissues, but the clinical significance of G9A expression in GC has rarely been studied. In this study, our investigations showed that G9A was highly expressed in GC tissues, and G9A expression was higher in metastatic GC than in non-metastatic GC. Furthermore, G9A expression significantly correlated with lymph node involvement and TNM stage, thus indicating that cancer cells expressing high levels of G9A exhibit a more invasive phenotype. Moreover, G9A expression significantly correlated with shorter survival and was an independent risk factor in patients with GC. In summary, G9A may be a novel prognostic marker of GC. Similar to some of our experimental results, Lin et al. found that increased G9A expression in GC decreased cell apoptosis and increased proliferation in GC cells^23^.

Peritoneal metastasis is the most common type of metastasis in advanced GC, and peritoneal metastasis indicates pathological stage IV disease, which is associated with a poor prognosis. Therefore, the early effective prevention and treatment of peritoneal metastasis are extremely important to improve the prognosis of patients with GC. Peritoneal metastasis is a sequential process that consists of three consecutive steps: highly malignant GC cells break through the serosa and enter the abdominal cavity, free cancer cells adhere to the peritoneum, and cancer cells form new capillaries and proliferate around the periphery of blood vessels^24,25^. Unfortunately, we were not able to measure G9A expression in peritoneal metastasis sites, because patients with GC and peritoneal metastases generally are no longer suitable candidates for curative surgery. Thus we suppressed and elevated G9A expression in GC cell lines and performed in vitro and in vivo functional assays to evaluate the biological significance of increased G9A expression in GC. Because cells break through the serosal layer in the first step of peritoneal metastasis, we investigated the role of G9A in GC cell migration and invasion. Cells that expressed higher levels of G9A were more motile, thus laying the foundation for peritoneal metastasis. Newly arrived tumor cells in the abdominal cavity experience anoikis and anchorage-independent growth, and the results of anoikis assays and soft agar assays showed the importance of G9A in promoting anoikis resistance and anchorage-independent growth. To survive, free cancer cells must adhere to the peritoneum. Thus Matrigel, several ECM components, and nude mouse peritoneum tissue were used to study the adhesion of tumor cells, and the results showed that G9A promoted adhesion of GC cells. In vivo, G9A promoted peritoneal metastasis in the mouse abdominal cavity, in agreement with the results obtained in vitro.

In addition, G9A can regulate gene transcription by modifying H3K9 and H3K27 methylation at the promoter region of some genes, in a manner dependent on its histone methyltransferase activity. The single/double methylation of H3K9 (H3K9me1/2) and H3K27 (H3K27me) increases the expression of suppression factors, such as HP1 family members, thus resulting in closer binding between DNA and histone proteins and consequently inhibiting gene transcription^17,18^. Alternatively, G9A can directly control the level of DNA methylation via DNA methyltransferases (DNMTs). Multiple cell experiments have shown that DNMT1 and G9A together maintain the transfer of methylation information during DNA replication^26^. In addition, G9A can also increase the levels of DNMT3a and DNMT3b via the ANK domain, thus maintaining DNA methylation at the gene promoter and regulating gene silencing in a manner independent of the methylation enzyme activity of G9A^27^. Traditionally, G9A has been believed to primarily methylate histones on regular chromosomes to subsequently inhibit the transcription of related genes. However, the latest research shows that G9A can also serve as a scaffold protein that increases the levels of transcriptional activators that activate the transcription of specific genes. Specifically, G9A interacts with multiple transcriptional activators and thereby co-activates gene transcription. Studies have reported that G9A, together with p300, CARM1, and GRIP1, as a coactivator for nuclear receptors in a methyltransferase activity-independent manner^28,29^. In human lung cancer cells, G9A recruits CARM1 and p300 to the promoter of the GR target genes ENaCα and CDH16 and promotes the transcription of these genes under the stimulation of glucocorticoids^19^. In these processes, the role of G9A in the activation of gene transcription does not depend on the methylation enzyme activity but is instead related to the ability of its structural domain to recruit other transcriptional activators. ITGB3, a member of the integrin family and a GR target gene, promotes the peritoneal metastasis of GC by influencing the ability of GC cells to adhere to the peritoneum^22,30^. In this study, the knockdown of G9A inhibited the DEX-induced expression of ITGB3, and silencing of ITGB3 attenuated the ability of G9A to promote invasion and adhesion in GC cells. Under DEX stimulation, G9A, P300, and GR formed a transcriptional activator complex, which in turn promoted the expression of ITGB3. ITGB3 was downregulated in G9A-silenced cells, and this effect was reversed after introduction of the G9A-△SET and G9A-mut vectors. This finding further demonstrated that G9A activates gene expression by interacting with nuclear receptors, and this activity is independent of G9A’s methyl transferase activity. Reg IV has been found to be associated with peritoneal metastasis in our previous study, and its overexpression upregulates many adhesion molecules, including ITGB3^21^. In this study, we found that Reg IV affects G9A expression by regulating the ERK/SP1 signaling pathway.

In conclusion, G9A does not rely on its methyltransferase activity to concomitantly activate the downstream effector ITGB3. We report the first evidence that the Reg IV-Erk-SP1-G9A complex (with GR and P300)-ITGB3 pathway contributes to GC development. Moreover, high G9A expression correlates with poor survival in patients with GC. These findings suggest that G9A may be an attractive target for suppressing GC metastasis.

## Materials and methods

### Specimens and IHC

The tissues used were obtained from the Shanghai Ruijin Hospital at the Shanghai Jiao Tong University School of Medicine. All patients provided written informed consent before enrollment, and the study protocol was approved by the Ethics Committee of Shanghai JiaoTong University School of Medicine of Ruijin Hospital. None of the patients had received chemotherapy or radiotherapy before surgery. All patients had curative intent and underwent radical resection. IHC was performed as previously described^31^, by using antibodies against G9A (R&D, Minneapolis, MN, USA). The percentage of the positive cancer cells and intensity of the cell staining were scored according to the following rules: Percentage of positive cells was classified as follows: <10% (0), 10–25% (1), >25–50% (2), >50–75% (3), and >75% (4). Intensity of staining was evaluated as negative (0), weak (1), moderate (2), or strong (3). The final scores of the tissue sections were multiplied by the intensity scores and percentage of positive cells scores: 0–2 final scores indicated negative expression, whereas 2–6 final scores represented weak positive expression and 6–12 final scores represented strong positive expression.

### Cell cultures and treatment

Human GC cell lines and human immortalized gastric epithelial cells were purchased from the Shanghai Institutes for Biological Sciences at the Chinese Academy of Sciences. The cells were maintained in 1640 medium (Gibco, Carlsbad, CA, USA) supplemented with 100 IU/ml penicillin, 100 IU/ml streptomycin, and 10% fetal bovine serum. The following inhibitors were used: BIX-01294 (Selleck, Houston, TX, USA) against G9A and PD98059 (Selleck) against ERK. All cells were incubated at 37 °C in a 5% CO_2_ and saturated humidity atmosphere.

### Establishment of stably transfected cells

The G9A shRNAs and overexpression lentivirus were purchased from the Novobio Biotechnology Company in Shanghai, China. The shRNA duplexes were synthesized as follows:

5′-CACCGAGAGAGTTCATGGCTCTTTCGAAAAAGAGCCATGAACTCTCTC -3′ (G9a shRNA 1 forward),

5′-AAAAGAGAGAGTTCATGGCTCTTTTTCGAAAGAGCCATGAACTCTCTC -3′ (G9a shRNA 1 reverse) and

5′-CACCGCATAGATGCCCGTTACTATGCGAACATAGTAACGGGCATCTATGC-3′ (G9a shRNA 2 forward),

5′-AAAAGCATAGATGCCCGTTACTATGTTCGCATAGTAACGGGCATCTATGC-3′ (G9a shRNA 2 reverse). G9A was knocked down or overexpressed by lentiviral infection, and 5 μg/ml blasticidin was used to select cells in which G9A was stably knocked down or overexpressed.

### Transfection of GC cells

GC cells were transfected with siRNA or shRNA with Lipofectamine 2000 according to the manufacturer’s protocol. The sense and anti-sense strands of siRNAs and shRNAs are listed in Supplementary Table S1.

### Total RNA isolation and qRT-PCR

Total RNA was extracted from GC cells and subjected to qRT-PCR as previously described^31^. Primers for qRT-PCR are listed in Supplementary Table S2.

### Western blot analysis

A western blot analysis was carried out as previously described^31^, with the following primary antibodies: anti-G9A (Biovision; Bioptics, Tucson, USA), anti-GAPDH (Abcam; Cambridge, MA, USA), anti-SP1 (Abcam), anti-p-SP1 (Abcam), anti-Flag (Sigma-Aldrich; St. Louis, MO, USA), anti-ERK1 (Cell Signaling Technology, Danvers, MA, USA), anti-phospho-ERK1/2(Cell Signaling Technology), anti-H3 (Cell Signaling Technology), anti-H3K9me2 (Cell Signaling Technology), anti-ITGB3 (Abcam), anti-GR (Abcam), and anti-P300 (Abcam).

### Wound-healing assay and Transwell assay

Wound-healing and Transwell assays were used to study changes in the migration and invasiveness of GC cells according to a previously described protocol^31^.

### Anoikis assay

GC cells undergoing anoikis were identified and quantified with a Cytoselect 24-well Anoikis Assay kit (Cell Biolabs; San Diego, CA, USA) according to the manufacturer’s instructions. Briefly, anoikis was detected by seeding 1 × 10^5^ cells in ultra-low attachment plates. After 48 h of culture, the cells were resuspended in 3-[4,5-dimethylthiazol-2-yl]-2,5 diphenyl tetrazolium bromide (MTT) and cell absorbance was detected at 570 nm.

### Adhesion assay

The adhesion of GC cells to ECM components was evaluated using a CytoSelect 48-Well Cell Adhesion Assay Kit (Cell Biolabs; San Diego, CA, USA) according to the manufacturer’s instructions. Matrigel or murine peritoneum was pre-coated in a 24-well plate, and GC cells labeled with carboxyfluorescein succinimidyl ester (AAT Bioquest; Sunnyvale, CA, USA) were overlaid on the plate bottom. Later, cells were incubated at 37 °C for 60 min and non-adherent cells were removed with three gentle washes with phosphate-buffered saline (PBS). The cells that were adherent to Matrigel or the murine peritoneum were then quantified in MTT assays or counted under a microscope, respectively.

### Soft agar colony-formation assay

GC cells in which G9A was knocked down or overexprssed were resuspended with 0.3% soft agar (Sigma-Aldrich; St. Louis, MO, USA) in RPMI 1640 containing 10% fetal bovine serum (FBS) and layered on 0.6% solidified agar in RPMI 1640 containing 10% FBS in six-well plates. The plates were incubated for 2 weeks. Colony sizes were photographed under a microscope at 10× magnification.

### ChIP assay

The ChIP assay was performed by using a ChIP kit (Millipore, Billerica, MA, USA) according to the manufacturer’s instructions. The resultant precipitated DNA samples were assessed with PCR.

### Luciferase reporter gene and Co-IPassays

The luciferase reporter gene and Co-IP assays were performed as previously described^32^.

### Animal experiments

All animal experiments were conducted according to the Chinese guidelines for animal experimentation and were approved by the Institutional Animal Care Committee of Ruijin Hospital, which is affiliated with the Shanghai Jiao Tong University School of Medicine. Briefly, 4-week-old male BALB/C nude mice were purchased from the Institute of Zoology at the Chinese Academy of Sciences of Shanghai. All experiments were performed in accordance with the official recommendations of the Chinese animal community. To establish the experimental peritoneal metastasis model, 1 × 10^6^ cells were resuspended in 0.1 ml of PBS and injected into the abdominal cavity. The mice were sacrificed after 80 days, and the number of nodules present in the abdominal cavity and the volume of ascites of each mouse were measured. During this time, we recorded the survival time of nude mice, and survival curves were generated. The organs were excised from nude mice post-mortem for hematoxylin and eosin staining.

### Statistical methods

The statistical analysis was performed with the SPSS Version 22.0 software (SPSS Inc, Chicago, IL, USA). Statistical analyses were performed using a one-way analysis of variance, Student’s *t*-test, chi-squared test, Fisher’s exact test, Cox proportional hazards regression, and the Kaplan–Meier method. *P* < 0.05 was considered significant, and *P* < 0.01 was considered highly significant.

## Electronic supplementary material

Supplementary figure legends

Supplementary tables

Figure S1

Figure S2

Figure S3

Figure S4

Figure S5

Figure S6

Figure S7
