# Supplementary material for: G9A promotes gastric cancer metastasis by upregulating ITGB3 in a SET domain-independent manner
Source: Cell Death Dis. 2018 Feb 15;9(3):278. doi: 10.1038/s41419-018-0322-6 (PMC5833452; doi:10.1038/s41419-018-0322-6)
Supplement: Supplementary file 1 — Supplementary figure legends [file 41419_2018_322_MOESM1_ESM.docx]

**Supplementary figure legends**

**Figure S1. Expression of G9A in GC cell lines and the effects of knockdown/overexpression lentiviruses.** (A) G9A mRNA expression in the Oncomine database. (B) G9A mRNA expression in the TCGA database. (C) G9A expression in four kinds of GC molecular subtypes. Among MSI and CIN groups, CIN and GS groups, EBV and GS groups, there were significant G9A expression differences. (D) G9A mRNA expression in six GC cell lines and one immortalized gastric epithelial cell line, as detected by qRT-PCR. (E-F) BGC-823/SGC-7901 and MKN-28/MKN-45 cells were transfected with sh-G9A lentivirus (E) and G9A overexpression lentivirus (F), respectively, and the mRNA levels were then detected by qRT-PCR. (G-H) SGC-7901 and MKN-45 cells transfected with sh-G9A lentivirus and G9A overexpression lentivirus, respectively, were subject to western blotting. Each value is the mean ± SD of three experiments. ***P*< 0.01.

**Figure S2. Effects of G9A on the wound healing, migration and invasion ability of GC cells (200×).** (A) Representative images of SGC-7901/sh-G9A cells, MKN-45/G9A cells and their control groups were recorded 0 and 48 hours after scratching of the cell surface. (B) The relative distances between the wound edges of GC cells at 0 and 48 hours. (C-D) The migration and invasive behavior were evaluated using Transwell chambers with or without Matrigel after the knockdown or overexpression of G9A in SGC-7901 or MKN-45 cells. (E) Histograms showed the average number of migrating cells in multiple fields. (F) Histograms showed the average number of invading cells in multiple fields. Each value is the mean ± SD of three experiments. ***P*< 0.01.

**Figure S3. G9A promotes soft agar colony formation in gastric cancer.** Each well of plates was added 500ul MTT solution and incubated in 37℃ for 1h. The pictures were captured with a digital camera. BGC-823/sh1 and BGC-823/sh2 groups showed smaller cloning than BGC-823/NC group, while MKN-28/G9A group showed bigger cloning than MKN-28/NC group.

**Figure S4. G9A promotes the peritoneal metastasis of GC cells.** (A) Anoikis assays of SGC-7901 and MKN-45 cells infected with a specific sh-G9A lentivirus and G9A overexpression lentivirus, as indicated. (B) Adhesion of G9A knockdown and overexpression GC cells to Matrigel-coated surfaces. (C-D) Percentage of knockdown (C) and overexpression (D) GC cells adherent to plates coated with different ECM components after 30 min of incubation were quantified on the basis of the OD at 560 nm. (E-F) Representative images (E) and number of cells (F) adherent to murine peritoneum after 60 min of incubation. (G-H) Representative images (G) and colony size of cells (H) in soft agar assay after 14 days of culture. Each value is the mean ± SD of three experiments. **P*< 0.05, ***P*< 0.01.

**Figure S5. G9A promotes peritoneal dissemination of GC cells in vivo.** Representative photos of tumor formation at the mesentery, diaphragm, liver and peritoneum are shown.

**Figure S6. Expression of SP1, ITGB3, GR and P300 in GC cell lines after transfection with specific siRNA or shRNA.** (A) SP1 mRNA and protein expression in MKN-28 cells after transfection with SP1 siRNA. (B) GR mRNA and protein expression in BGC-823 cells after transfection with GR shRNA. (C) P300 mRNA and protein expression in BGC-823 cells after transfection with P300 shRNA. (D) ITGB3 mRNA and protein expression in MKN-28/G9A cells after transfection with ITGB3 shRNA.

**Figure S7. G9A forms the GR/P300/G9A complex need DEX stimulation.** Without DEX stimulation, Co-IP signal was negative or weak among GR, P300 and G9A protein.
