# Supplementary material for: G9A promotes gastric cancer metastasis by upregulating ITGB3 in a SET domain-independent manner
Source: Cell Death Dis. 2018 Feb 15;9(3):278. doi: 10.1038/s41419-018-0322-6 (PMC5833452; doi:10.1038/s41419-018-0322-6)
Supplement: Supplementary file 2 — Supplementary tables [file 41419_2018_322_MOESM2_ESM.docx]

**Table S1: The Target sequences of siRNAs and shRNAs.**

| Gene | Target sequence |
| --- | --- |
| P300 shRNA-1 | CGGCAAACAGTTGTGCACA |
| P300 shRNA-2 | AGCTACTGAAGATAGATTA |
| GR shRNA-1 | GATGTGATGGACTTCTATA |
| GR shRNA-2 | TGACTGTAGCTGTAGGTGA |
| ITGB3 shRNA-1 | CCAGATGATTCGAAGAATT |
| ITGB3 shRNA-2 | GCAATGTCCTCCAGCTCAT |
| SP1 siRNA-1 | CCAACAGATTATCACAAAT |
| SP1 siRNA-2 | GGCTGGTGGTGATGGAATA |

**Table S2: Oligonucleotide sequence of qRT-PCR primers**

| Gene | Forward primer | Reverse primer |
| --- | --- | --- |
| G9A | 5’-CTGTCAGAGGAGTTAGGTTCTGC-3’ | 5’-CTTGCTGTCGGAGTCCACG-3’ |
| P300 | 5’-TTCCCCTAACCTCAATATGGGAG-3’ | 5’-GCCTGTGTCATTGGGCTTTTG-3’ |
| GR | 5’-ATAGCTCTGTTCCAGACTCAACT-3’ | 5’-TCCTGAAACCTGGTATTGCCT-3’ |
| SP1 | 5’-TGGCAGCAGTACCAATGGC-3’ | 5’-CCAGGTAGTCCTGTCAGAACTT-3’ |
| ITGB3 | 5’-GTGACCTGAAGGAGAATCTGC-3’ | 5’-CCGGAGTGCAATCCTCTGG-3’ |
| GAPDH | 5’-GGACCTGACCTGCCGTCTAG-3’ | 5’-GTAGCCCAGGATGCCCTTGA-3’ |

**Table S3: SP1 binding sites for G9A**

| **Start position** | **End position** | **Anchor position** | | **Strand** | **Sequence** |
| --- | --- | --- | --- | --- | --- |
| 171 | 187 | | 179 | + | ccacgGGGCggatgggg |
| 317 | 333 | | 325 | + | ggcgcGGGCggggcgcg |
| 370 | 386 | | 378 | + | ttgggGGGCgggcccga |
| 693 | 709 | | 701 | - | gggagGGGCgggggcgg |
| 813 | 829 | | 821 | + | ccccgGGGGggggtggg |
| 818 | 834 | | 826 | + | gggggGGGTggggaaaa |
| 931 | 947 | | 939 | + | cgtggGGGCagggcgtg |
| 1033 | 1049 | | 1041 | + | aaaggGGGCggggtgag |
| 1184 | 1200 | | 1192 | - | ctcggGGGCggggcagc |
| 1288 | 1304 | | 1296 | + | ccgggGGGCtgggcaag |
| 2314 | 2330 | | 2322 | + | ccgggGGGCggggggca |
| 2322 | 2348 | | 2340 | + | cctggGGGCggggggca |
| 4986 | 5002 | | 4994 | + | acccgGGGCggccgcgc |
