# Supplementary figures and images for: G9A promotes gastric cancer metastasis by upregulating ITGB3 in a SET domain-independent manner
Source: Cell Death Dis. 2018 Feb 15;9(3):278. doi: 10.1038/s41419-018-0322-6 (PMC5833452; doi:10.1038/s41419-018-0322-6)

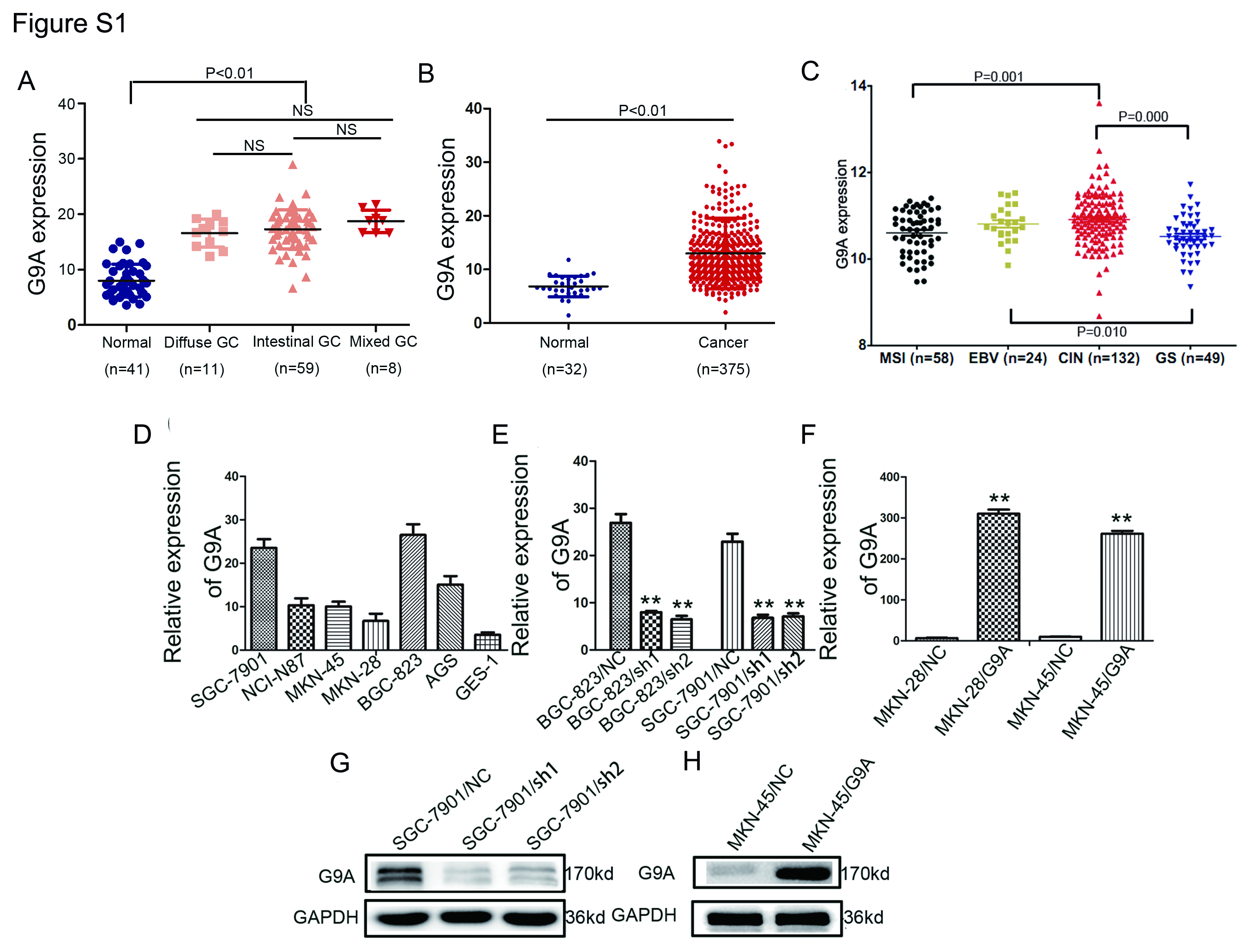

Supplement: Supplementary file 3 — Figure S1 [file 41419_2018_322_MOESM3_ESM.tif]

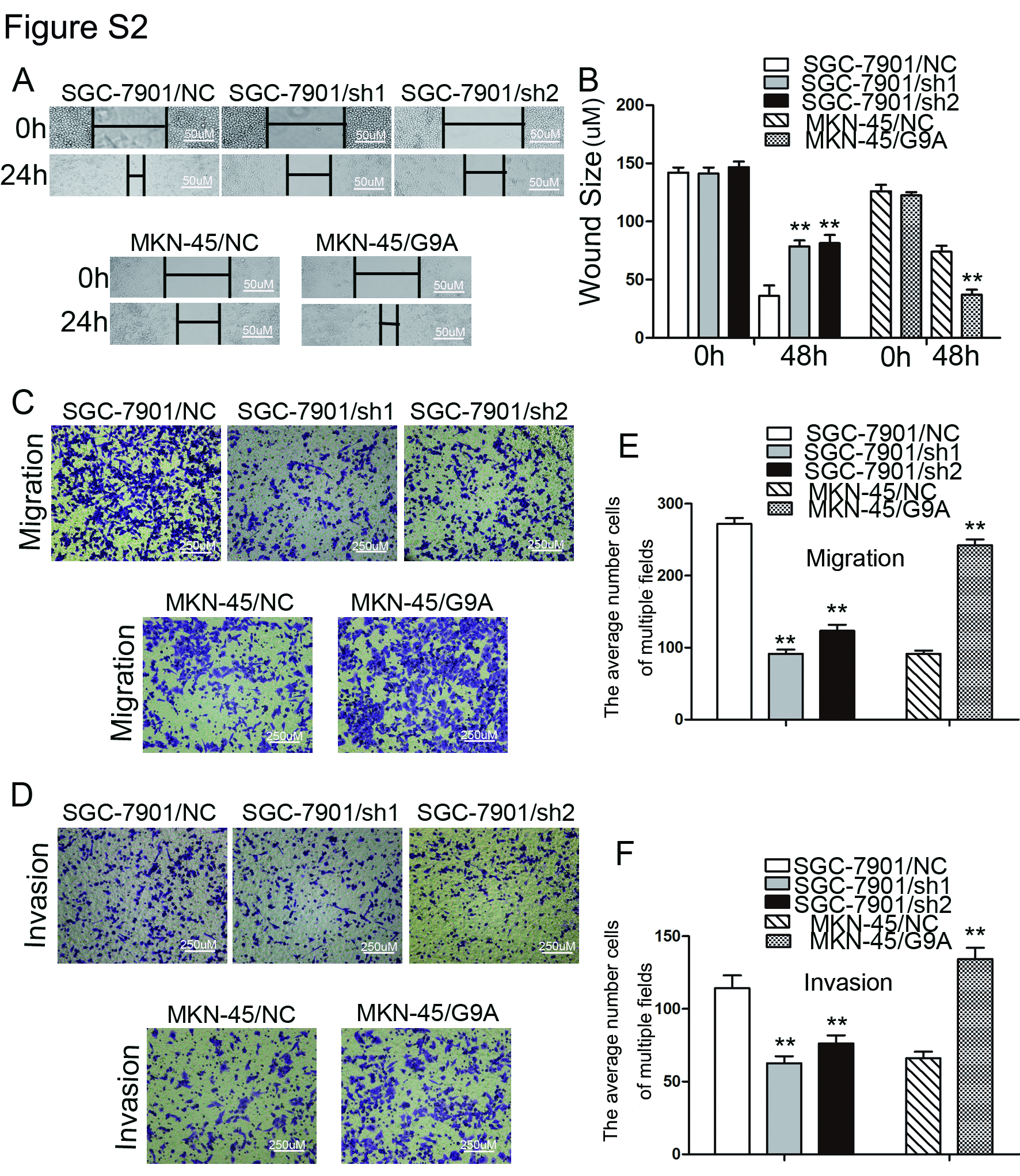

Supplement: Supplementary file 4 — Figure S2 [file 41419_2018_322_MOESM4_ESM.tif]

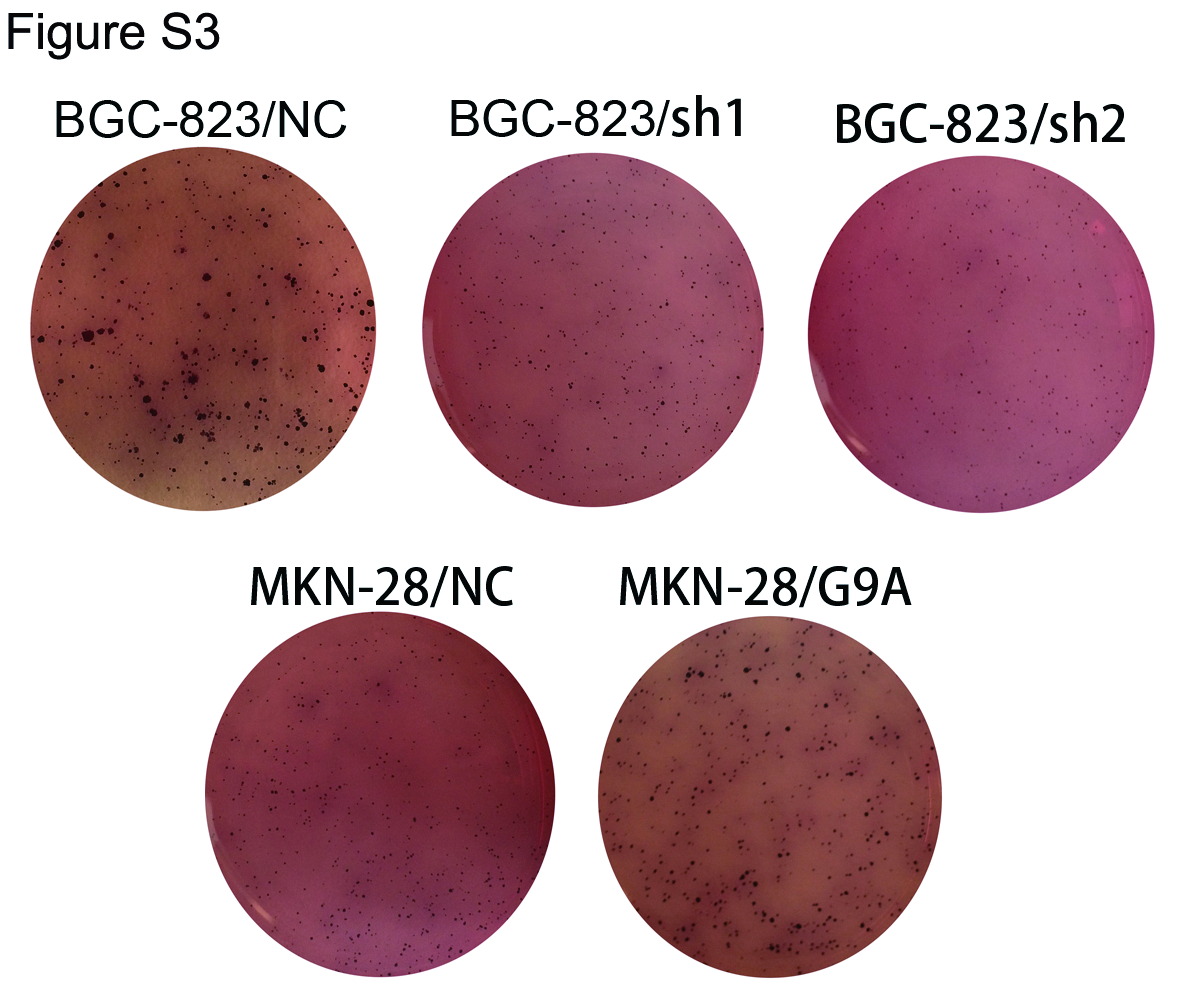

Supplement: Supplementary file 5 — Figure S3 [file 41419_2018_322_MOESM5_ESM.tif]

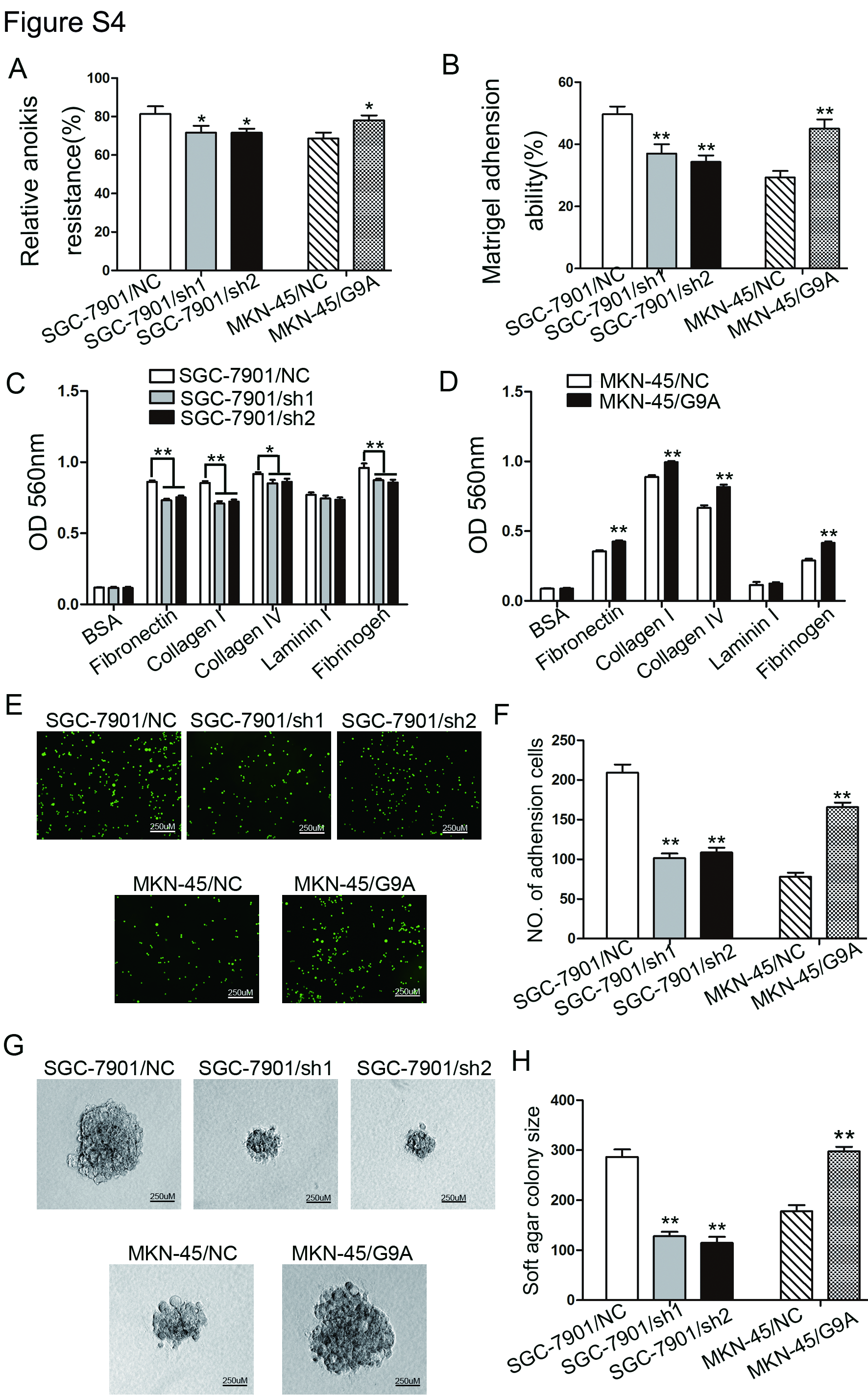

Supplement: Supplementary file 6 — Figure S4 [file 41419_2018_322_MOESM6_ESM.tif]

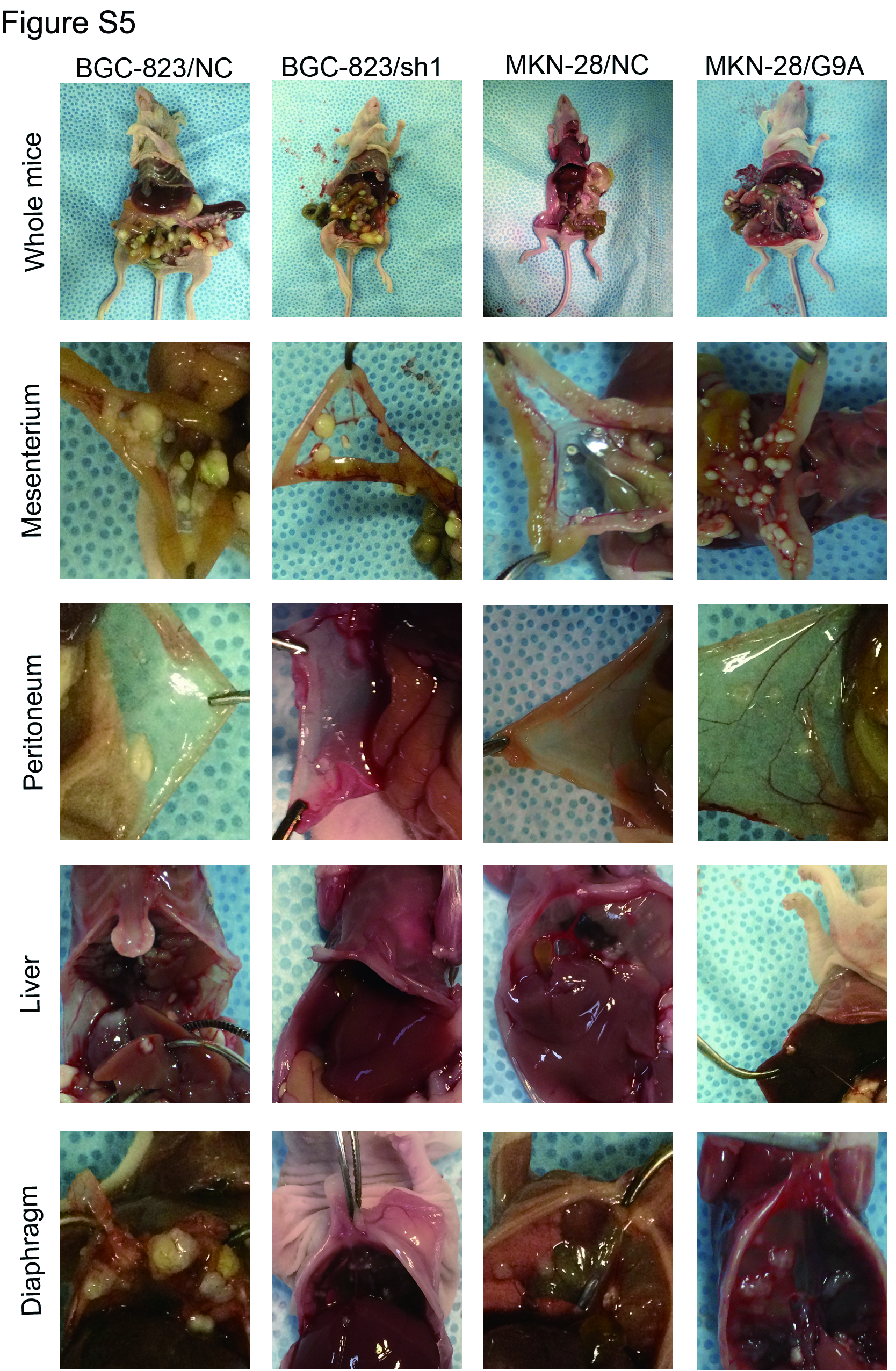

Supplement: Supplementary file 7 — Figure S5 [file 41419_2018_322_MOESM7_ESM.tif]

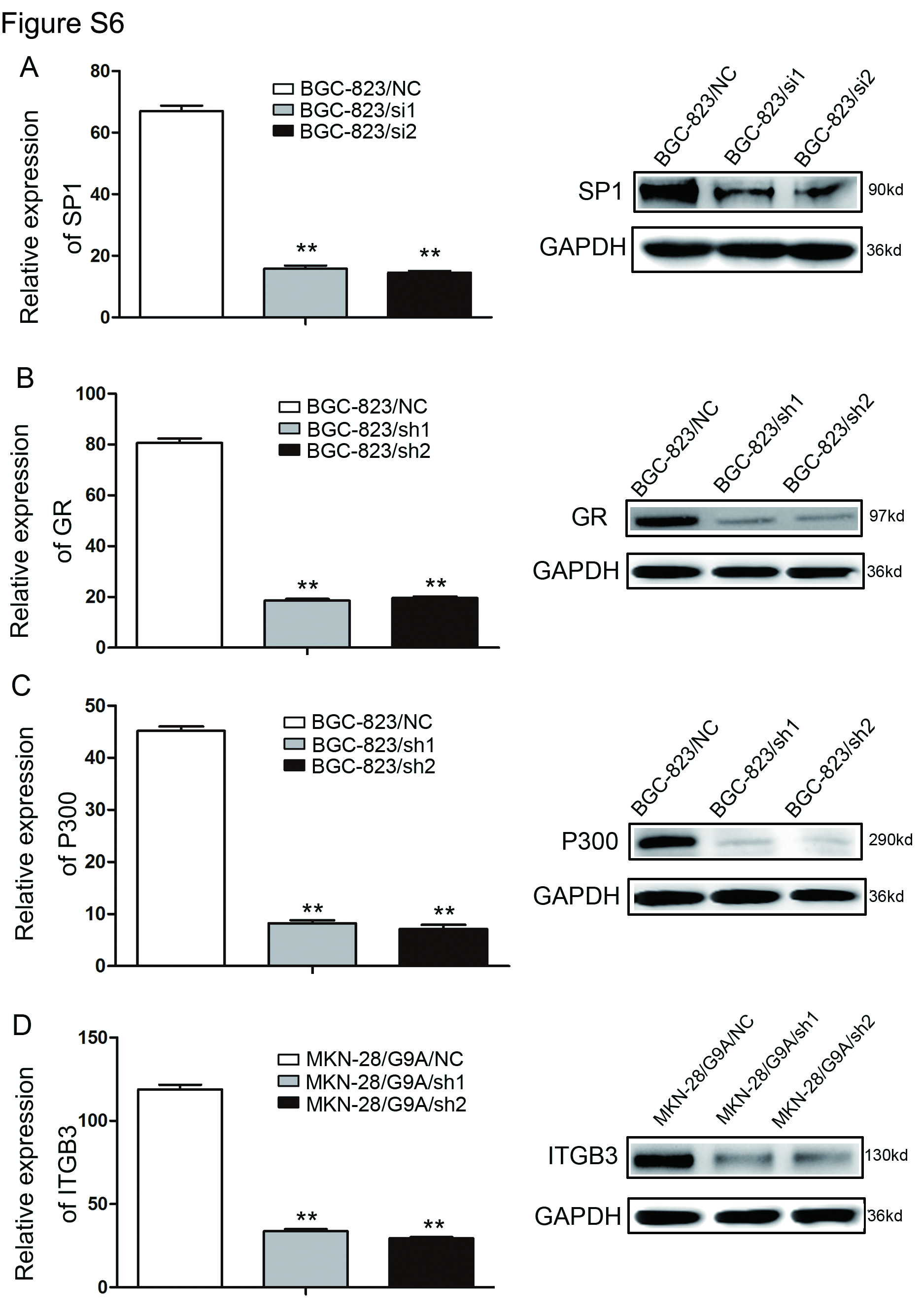

Supplement: Supplementary file 8 — Figure S6 [file 41419_2018_322_MOESM8_ESM.tif]

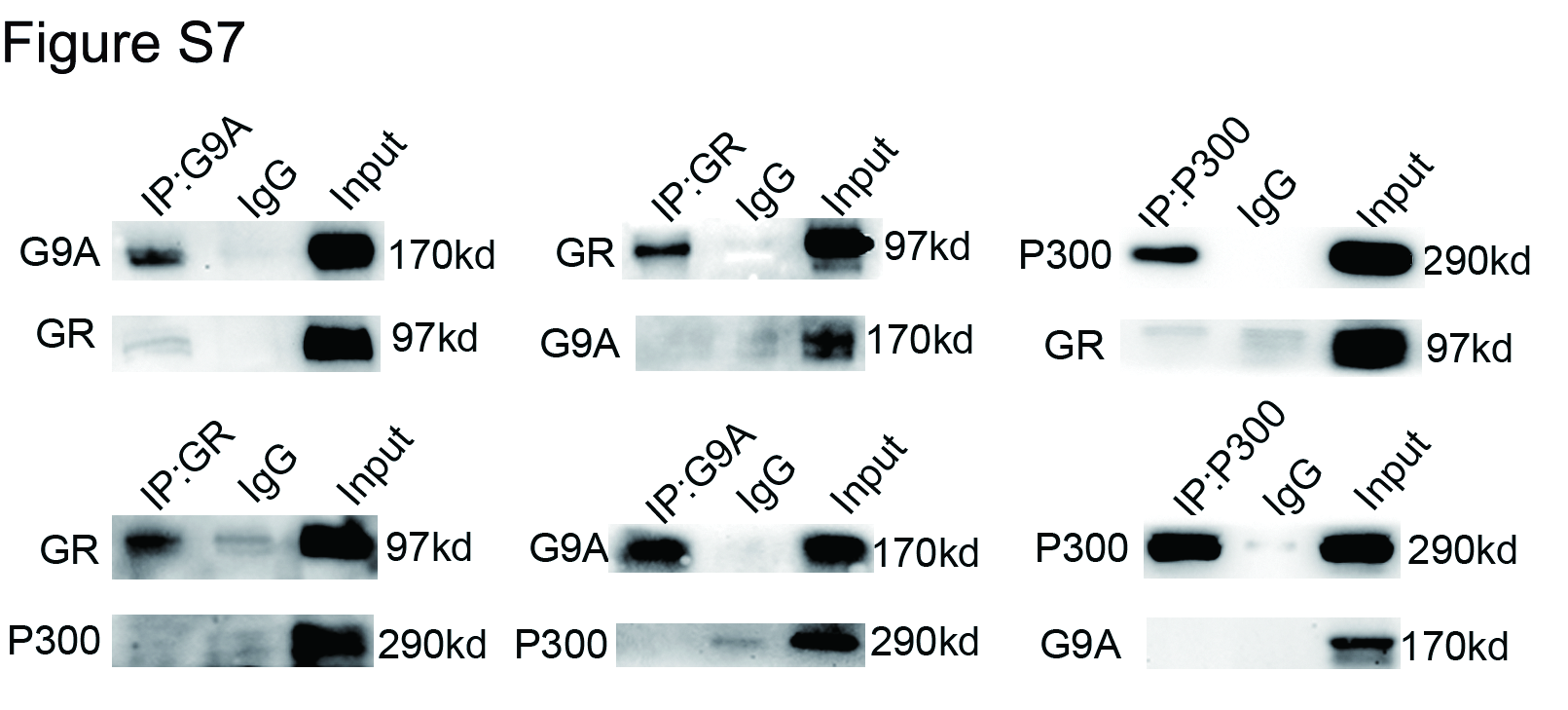

Supplement: Supplementary file 9 — Figure S7 [file 41419_2018_322_MOESM9_ESM.tif]
